# Prevalence and associated factors of vision loss in the South African National Health and Nutrition Examination Survey (SANHANES-1)

**DOI:** 10.1186/s12886-020-01714-4

**Published:** 2021-01-01

**Authors:** Emmanuel Kofi Addo, Kwadwo Owusu Akuffo, Ronel Sewpaul, Natisha Dukhi, Eldad Agyei-Manu, Akosua Kesewah Asare, David Ben Kumah, Moses Awuni, Priscilla Reddy

**Affiliations:** 1 Department of Optometry and Visual Science, College of Science, Kwame Nkrumah University of Science and Technology, Kumasi, Ghana; 2 Department of Ophthalmology and Visual Sciences, John A. Moran Eye Centre, University of Utah, Salt Lake City, Utah USA; 3 Department of Nutrition and Integrative Physiology, University of Utah, Salt Lake City, Utah USA; 4 Health & Wellbeing, Human and Social Capabilities Division, Human Sciences Research Council, Cape Town, South Africa; 5 Usher Institute for Population Health Sciences and Informatics, College of Medicine and Veterinary Medicine, University of Edinburgh, Edinburgh, UK; 6 Faculty of Health Sciences, Nelson Mandela University, Port Elizabeth, South Africa

**Keywords:** Vision loss, Prevalence, SANHANES, Associated factors/determinants, Barriers, Disparities, Eyecare services, South Africa

## Abstract

**Background:**

Vision loss is a major public health concern that significantly affects developing countries, including South Africa. Although existing literature have reported on the prevalence, causes, and impact of vision loss on the quality of life of affected individuals (children and adults) in parts of South Africa, there is no evidence of the prevalence and associated factors of vision loss in the general population. Hence, this study aimed to determine the prevalence of vision loss and its associated factors in South Africa using a population-based survey.

**Methods:**

Secondary analyses were conducted using data from the South African National Health and Nutrition Examination Survey (SANHANES-1), a population-based national health survey conducted from 2011 to 2012. Vision loss was defined as presenting visual acuity (PVA) worse than Snellen 6/12 in the better eye. Visual acuity was assessed by clinicians and participants’ subjective response to vision-related questions. Univariate and multiple logistic regression models were used to examine the association of the independent variables with vision loss.

**Results:**

The analytic sample comprised 4346 individuals with a mean age of 39.1 years. Female sex accounted for 55.6% of the participants. The prevalence of vision loss among participants was 9.2% (95% CI: 7.7–10.9). Older age (45–54 years, OR = 2.99, *p* < 0.001; 55–64 years, OR = 5.78, *p* < 0.001 and ≥ 65 years, OR = 5.12, *p* < 0.001), female sex (OR = 1.50, *p* = 0.016), and previous diabetes diagnosis (OR = 2.28, *p* = 0.001) were significantly associated with increased odds of vision loss. Further, secondary school education (OR = 0.71, *p* = 0.031), white ethnicity (OR = 0.11, *p* = 0.007), residing in Mpumalanga province (OR = 0.12, *p* < 0.001) and having never had an eye examination (OR = 0.56, *p* = 0.003) were significantly associated with reduced odds of vision loss.

**Conclusion:**

Almost one in ten participants had vision loss. Adopting strategies targeted at reducing barriers to the utilization of eye care services will promote early detection and management of blinding conditions, and thereby, decrease the burden of vision loss in South Africa.

## Background

Vision loss (visual impairment and blindness) is a major public health concern worldwide [1–3] as it negatively impacts the quality of life [4–6], employment prospects, and socioeconomic status [4–6], and also, increases depression and anxiety in the elderly [7, 8]. In children, vision loss limits potential maximization as it affects motor skills and emotional development [9], self-image [10, 11], academic prowess [12, 13], and social relations [14]. These challenges affect productivity, economic development, and also places an extreme burden on the country’s healthcare systems [15, 16]. Recent systematic review and meta-analysis estimates that about 253 million people in the world have vision loss; 36 million of which are blind, with 217 million having moderate or severe visual impairment [1–3]. Uncorrected refractive error (49%) and cataract (26%) are the principal causal factors of vision loss globally [1–3]. Notably, gender, age, and economic status influence the prevalence of vision loss worldwide. Thus, women (55%), adults aged 50 years and above (80%), and people living in developing countries (90%) have an increased prevalence of vision loss [1, 2]. A systematic review and meta-analysis published on the global burden of eye disease reports a prevalence of 2.90% of visual impairment and 0.48% of blindness in the world [1].

The distribution of vision loss is affected by both nonmodifiable risk factors (such as aging [17–19], genetics [20, 21], and ethnicity [18]) and modifiable risk factors (such as lifestyles [22, 23], and nutrition [24]). Recent progress in healthcare delivery has led to an upsurge in the life expectancy of the population worldwide [25]. Aging causes various structural and physiological changes that predispose adults to ocular morbidity (such as presbyopia, cataract, glaucoma, and age-related macular degeneration) [17–19]. Genetics has been shown to play a vital role in the etiology of some visual impairments. For instance, a positive family history of glaucoma and retinitis pigmentosa increases the risk of one developing the same conditions [20, 21]. Also, ethnicity has been reported to be associated with vision loss; individuals of African descent are at an increased risk of developing glaucoma than Caucasians [18, 26]. Again, smoking has been shown to increase the risk of developing cataract [23] or causing early-onset or rapid progression of age-related macular degeneration (AMD) [22]. Additionally, reduced access to good nutrition is implicated in the development of visual impairment. For instance, inadequate intake of foods rich in micro-nutrients such as Vitamin A could lead to xerophthalmia, a type of Vitamin A deficiency that presents with symptoms of night blindness, and eventually blindness from corneal scarring when untreated [24]. Besides these factors, it is worth pointing out that socioeconomic status and demographic factors play a crucial role in the burden of vision loss [27].

Africa represents about 12% of the world’s population; however, it contributes almost a fifth to the global burden of vision loss [28, 29]. This heightened burden is occasioned by the perennial challenge of inadequate eye care professionals and their disproportionate distribution across nations, and the absence of eye care facilities in the much needed rural areas [30]. Southern sub-Saharan Africa comprising of five countries (including South Africa) have a crude prevalence of vision loss of 2.11%, with the leading causes being uncorrected refractive error (0.84%) and cataract (0.59%) [1]. Previously, the National Guideline on Prevention of Blindness estimated a 0.75% prevalence of blindness in South Africa [31]. However, a recent study by Naidoo et al. reported a 0.90% prevalence of blindness; with cataract, refractive error, and glaucoma being the leading causes of vision loss in South Africa [32]. Of note, 80% of the population of South Africa suffer from extreme poverty, with the majority of the blind (80%) living in rural communities [31].

Although existing literature have reported on the prevalence of visual loss/visual impairment in different districts/ provinces in South Africa, there is no evidence of the prevalence and associated factors of vision loss at the national level [31, 32]. Furthermore, a recent systematic review and meta-analysis by Naidoo et al. in 2020 showed that about half of the countries that constitute the sub-Saharan Africa region have no nationally representative data on the prevalence of vision loss [33]. Therefore, this study seeks to determine the prevalence of vision loss and its associated factors in South Africa using data from the South African National Health and Nutrition Examination Survey (SANHANES-1). This study will also inform policymakers, healthcare administrators, and eye care professionals on the need to revise public health policies, and further, promote efficient and equitable allocation of resources to alleviate the burden of vision loss in South Africa and sub-Saharan Africa at large.

## Methods

### Study design

Secondary analyses were conducted on data from the South African National Health and Nutrition Examination Survey (SANHANES-1), a population-based bio-behavioural national survey conducted in 2011–2012 [34]. Data collection comprised interviews, general physical examination and biomarker analyses. Additional details of SANHANES-1 methodology and laboratory procedures are reported by Shisana et al. [34].

### Sociodemographic characteristics by province

South Africa (SA) has nine (9) provinces, namely: Gauteng, KwaZulu-Natal, Western Cape, Eastern Cape, Limpopo, Mpumalanga, North West, Free State and Northern Cape. As of 2019, SA had a mid-year population estimates of 58.78 million with female sex accounting for nearly 30 million (51.2%). Most of the SA population, about 15.2 million (25.8%) and 11.3 million (19.2%) resides in Gauteng (smallest province and yet highly urbanized) and KwaZulu-Natal respectively, with Northern Cape being the smallest province with a population of 1.26 million (2.2%). Furthermore, Gauteng, Western Cape and KwaZulu-Natal provinces have large metropolitan urban areas, and together account for 58% of the national population. Mpumalanga, North West, Limpopo, Free State, Northern Cape, and Eastern Cape contain large rural areas. Mpumalanga province has a large rural population and has the second highest provincial prevalence of adults with no formal education. In addition, about 28.8% of the SA population are younger than 15 years and mainly reside in Gauteng and KwaZulu-Natal. Of note, nearly 9% of the population are aged 60 years and above. Moreover, a third (17.8 million) of the SA population comprises the youth (aged 18–34 years), with nearly half residing in Gauteng (28.6%) and KwaZulu-Natal (19.4%). The Free State (4.7%) and the Northern Cape (2.0%) have the lowest proportions of youth [35].

### Sampling

**Fig. 1:**
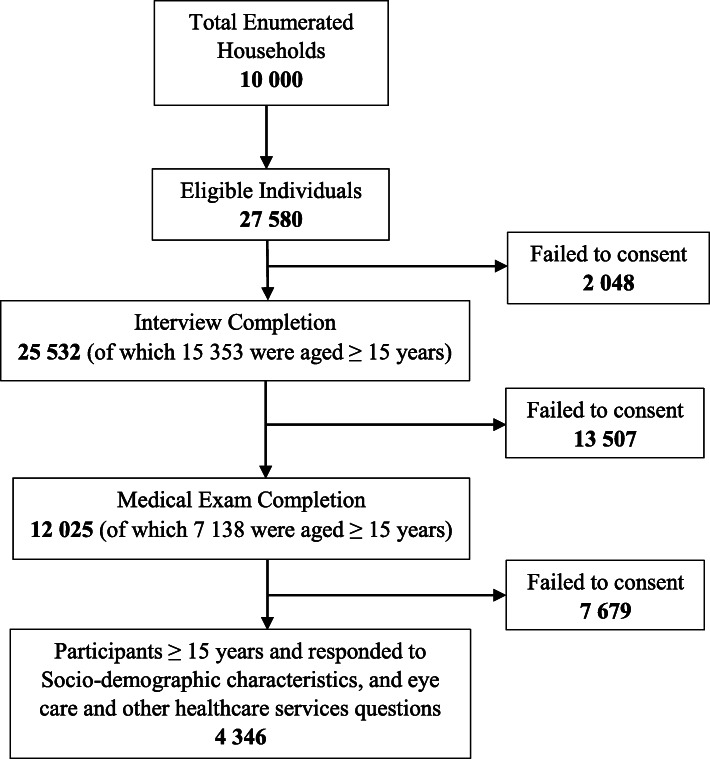
Flowchart showing the derivation of the analytic sample

Multi-stage disproportionate, stratified cluster sampling was used to select households within enumeration areas (EAs) stratified by province and locality type. A total of 10,000 households were selected. Within the occupied households, 27,580 individuals of all ages were eligible to be interviewed and agreed to participate; out of whom 25,532 (92.6%) completed the interview. Of the latter number, 12,025 (43.6%) individuals volunteered to undergo medical examination**.** A total of 15,353 participants aged ≥15 years completed the interview of which 7138 individuals aged ≥15 years underwent a physical examination. Furthermore, 2048 of the eligible individuals did not consent to being interviewed, 13,507 of those who completed the interview did not agree to undergo a physical examination and 7679 of those who completed the physical examination did not answer the questions on eye care and other healthcare services and thus, were excluded from this study (see Fig. 1).

### Visual examination

Trained survey staff administered the interviews and clinical teams each comprising a registered nurse, a medical doctor, and clinical assistant, conducted the physical examinations. During the physical examination, the medical doctor assessed participants’ visual acuity (using a Snellen chart) to determine whether participants had vision loss. Vision loss was defined as presenting visual acuity (PVA) worse than Snellen 6/12 in the better eye. Participants with vision loss were subsequently asked to provide self-reports on the kind(s) of vision-related difficulty they experienced; with response options being blurred vision, a need for more light, difficulty reading, loss of peripheral vision, difficulty driving at night, double vision, difficulty in distinguishing colours, straight lines looking wavy, and sensitivity to glare. In this study, ‘blurred vision’ was defined as participants’ self-reported symptoms of inability to see objects sharply, especially during the execution of their normal day-to-day activities. ‘A need for more light’ encapsulates all forms of ocular pathology and refractive conditions that necessitate the need for an additional source of light to enable optimal visual functioning. ‘Difficulty reading’, ‘loss of peripheral vision’, ‘difficulty driving at night’, ‘double vision’, ‘difficulty in distinguishing colours’, ‘straight lines looking wavy’, and ‘sensitivity to glare’ were all based on participants’ self-reported vision difficulty. The categories were not mutually exclusive, that is, a participant could experience multiple types of vision difficulties. All doctors employed in the study were trained in standardised procedures of measuring visual acuity.

### Ethical approval

Ethical approval for the study was obtained from the Research Ethics Committee (REC) of the South African Human Sciences Research Council (HSRC) (REC number: 6/16/11/11). The study adhered to the tenets of the Declaration of Helsinki. Written informed consent/assent was obtained from all the survey participants. Written informed consent was also obtained from the parents or legal guardians of participants under the age of 18 years.

### Measures

The primary outcome variable was vision loss, assessed by the medical doctor during the physical examination. Vision loss was defined as PVA worse than Snellen 6/12 in the better eye [36]. Independent variables, obtained from the interview, were sex, age group, ethnicity, education level, employment status, urban residence, province, diabetes diagnosis, and the number of years since their last eye examination. Ethnicity was reported according to Statistics South Africa’s standard classification groups [37]. These variables were investigated because they have been found to be associated with vision loss in several studies [1, 2, 17–19, 32, 38–43]. In addition, age, sex, ethnicity and education have been found to confound the relationship between diabetes diagnosis and healthcare seeking on vision loss [42–46].

### Data analysis

Data were analysed in Stata 15.0. (StataCorp, Texas, USA 2016). The analyses applied sample weights to adjust for unequal probabilities of selection and nonresponse. A total of 4346 individuals aged ≥15 years underwent a physical examination and responded to the interview questions on socio-demographic characteristics and the use of eye care and other healthcare services. The prevalence of vision loss was presented by categories of the independent variables, and pairs of estimates were considered statistically different if their 95% confidence intervals did not overlap. Among those who were assessed to have vision loss, the prevalence of the three most reported vision difficulties in this study, namely, blurred vision, a need for more light, and difficulty reading, were presented by the independent variables. Univariate and multiple logistic regression models were used to examine the association of the independent variables with the primary outcome; vision loss. Only the variables that were found to be significant in the univariate logistic regression were included in the multiple logistic regression model.

## Results

### Description of the sample

The mean age of all participants was 39.1 years (see Table 1). Two-thirds of the sample had a secondary school education, and 10% had tertiary education. Six percent (6%) reported that they had been previously diagnosed with diabetes. Nearly a third of the participants (31.1%) had ever had an eye examination, with 19.5% having had an eye examination within the last 2 years.

**Table 1:** Description of the sample

	n	%
Total	4346	100.0
Age (mean, S.D.)	39.1	18.1
15–44	2712	72.1
45–54	671	13.1
55–64	527	8.2
≥ 65	436	6.6
Sex		
Males	1541	44.4
Females	2805	55.6
Ethnicity		
African	3054	78.4
White	87	8.4
Mixed race	995	10.4
Indian/Asian	210	2.8
Highest Education		
No formal schooling/Gr0–7	1477	23.9
Grade 8–12 (or equivalent)	2624	66.0
Higher education	245	10.0
Employed		
No	3263	71.4
Yes	1083	28.6
Urban/Rural residence		
Rural	1722	37.5
Urban	2624	62.5
Province		
Western Cape	749	14.0
Eastern Cape	594	12.7
Northern Cape	326	2.8
Free State	309	5.5
KwaZulu Natal	678	19.4
North West	382	5.1
Gauteng	414	21.7
Mpumalanga	518	7.8
Limpopo	376	11.1
Self-reported diagnosis of diabetes		
No previous diabetes diagnosis	4030	94.0
Previous diabetes diagnosis	316	6.0
Years since last eye examination		
≤ 2 years	761	19.5
3–5 years	266	6.2
> 5 years	172	5.4
Never	3147	68.9
Do you use eyeglasses or contact lenses to see far away^a^		
Yes	468	13.8
No	3852	86.2
Do you use eyeglasses or contact lenses to see up close^a^		
Yes	600	17.3
No	3699	82.7
In the last 30 days, how difficult was it to see & recognize an object or a person you know across the road (from a distance of about 20 m)?^a^		
None	3367	83.8
Mild	408	8.1
Moderate	270	5.0
Severe	162	3.1
In the last 30 days, how difficult was it to see & recognize an object at arm’s length^a^		
None	3348	83.3
Mild	452	8.7
Moderate	256	5.0
Severe	148	3.0

n, frequency; %, percentage of the frequency; ^a^totals per category do not always add to overall total due to missing data and non-response (the number of missing observations for the last 4 variables; using visual aids to see far away, using visual aids to see up close, difficulty in seeing objects from across the road and difficulty in seeing objects at arm’s length; were 26, 47, 139, and 142 respectively)

### Prevalence of vision loss

Almost one in ten people (9.2 95% CI: 7.7–10.9) were classified as having vision loss (see Table 2). Significantly more adults aged 45–54 years (16.9%), 55–64 years (23.4%) and ≥ 65 years (21.1%) experienced vision loss than those aged 15–44 years (5.1%). Vision loss was significantly higher among participants of Indian ethnicity (29.5%) than in African (9.3%), mixed-race (8.8%), and white (2.2%) ethnicities. Participants who had not completed secondary school (15.1%) had significantly higher prevalence of vision loss than those who completed secondary (7.5%) and tertiary (6.3%) education. KwaZulu-Natal province had the highest prevalence of vision loss (17.6%) whereas, Mpumalanga province had the lowest (1.3%). Vision loss was more than four times higher in participants who were diagnosed with diabetes (30.1%) than those who were not diagnosed with diabetes (7.8%). Vision loss was lower in participants who had never had an eye examination (7.7%) than in those who had an eye examination within the preceding 2 years (13.3%).

**Table 2:** Prevalence of vision loss

	Vision loss		
	frequency	%	95% CI
Total	481	9.2	[7.7–10.9]
Age (years)			
15–44	111	5.1	[3.8–6.8]
45–54	117	16.9	[12.4–22.5]
55–64	140	23.4	[17.0–31.3]
≥ 65	113	21.1	[14.8–29.3]
Sex			
Males	160	7.2	[5.6–9.1]
Females	321	10.8	[8.8–13.2]
Ethnicity			
African	290	9.3	[7.5–11.3]
White	3	2.2	[0.7–7.0]
Mixed race	118	8.8	[5.9–12.7]
Indian/Asian	70	29.5	[21.2–39.3]
Highest Education			
No formal schooling/Gr0–7	240	15.1	[12.1–18.6]
Grade 8–12 (or equivalent)	215	7.5	[5.9–9.4]
Higher education	26	6.3	[3.4–11.4]
Employed			
No	377	9.8	[8.0–11.9]
Yes	104	7.7	[5.5–10.7]
Urban/Rural residence			
Rural	155	8.3	[6.4–10.7]
Urban	326	9.7	[7.7–12.2]
Province			
Western Cape	87	7.8	[5.2–11.7]
Eastern Cape	36	6.3	[4.1–9.6]
Northern Cape	34	5.8	[3.2–10.4]
Free State	41	9.9	[5.3–17.8]
KwaZulu Natal	165	17.6	[13.2–23.1]
North West	26	5.1	[2.8–9.2]
Gauteng	54	9.2	[5.6–14.7]
Mpumalanga	9	1.3	[0.5–3.4]
Limpopo	29	7.3	[4.3–12.0]
Self-reported diagnosis of diabetes			
No previous diabetes diagnosis	386	7.8	[6.5–9.5]
Previous diabetes diagnosis	95	30.1	[22.4–39.3]
Years since last eye examination			
≤ 2 years	158	13.3	[9.7–18.0]
3–5 years	38	13.2	[8.1–20.9]
> 5 years	27	8.8	[4.6–16.4]
Never	258	7.7	[6.2–9.4]
Do you use eyeglasses or contact lenses to see far away†			
Yes	111	14.3	[9.3–21.2]
No	367	8.4	[7.0–10.1]
Do you use eyeglasses or contact lenses to see up close†			
Yes	126	12.4	[8.2–18.2]
No	348	8.5	[7.1–10.2]
In the last 30 days, how difficult was it to see & recognize an object or a person you know across the road (from a distance of about 20 m)?†			
None	269	6.3	[5.1–7.7]
Mild	84	19.1	[13.0–27.0]
Moderate	63	24.1	[17.7–31.8]
Severe	49	36.3	[23.0–52.0]
In the last 30 days, how difficult was it to see & recognize an object at arm’s length†			
None	260	6.3	[5.1–7.7]
Mild	98	18	[13.1–24.2]
Moderate	62	23.4	[16.9–31.4]
Severe	46	40	[26.0–55.8]

%, percentage of frequency; *CI* Confidence Interval

The three most prevalent types of vision difficulties reported by participants who were found to have vision loss were difficulty reading (56.2%), blurred vision (42.3%), and a need for more light (18.3%) (see Table 3). Reading difficulty was significantly more prevalent among 45–54-year olds (74.9%) than 15–44-year olds (45.5%). Blurred vision was significantly higher among rural residents (61.5%) than urban (32.5%) residents. The prevalence of reporting a need for more light did not differ significantly between categories of the independent variables.

### Factors associated with vision loss

The univariate logistic regression analysis showed that older age (45–54 years, odds ratio (OR) = 3.79, *p* < 0.001, 55–64 years, OR = 5.71, p < 0.001 and ≥ 65 years, OR = 5.00 *p* < 0.001 compared with 15–44 years), female sex (OR = 1.57, *p* = 0.003), Indian ethnicity (OR = 4.09, *p* < 0.001 compared with African ethnicity), residing in KwaZulu-Natal province (OR = 2.52, *p* = 0.001 compared with Western Cape province) and previous diabetes diagnosis (OR = 5.07, *p* < 0001) were significantly associated with experiencing vision loss (see Table 4). White ethnicity (OR = 0.23, *p* = 0.014 compared with African ethnicity), higher education levels (secondary schooling, OR = 0.46, *p* < 0.001 and tertiary education, OR = 0.38, *p* = 0.006 compared with primary school or no formal schooling), residing in Mpumalanga province (OR = 0.16, *p* = 0.001 compared with Western Cape province) and having never had an eye examination (OR = 0.54, p = 0.001 compared with having had an eye examination within the preceding 2 years) were significantly associated with reduced odds of vision loss.

**Table 3:** Self-reported vision difficulties among participants who had vision loss

	Blurred vision			Need for more light			Difficulty reading			Other difficulties^b^		
	n	%	95% CI	n	%	95% CI	n	%	95% CI	n	%	95% CI
Total	203	42.3	[33.9–51.2]	84	18.3	[12.5–25.9]	274	56.2	[46.8–65.1]	86	21.1	[15.9–27.4]
Age (years)												
15–44	38	40.1	[26.8–54.9]	11	11.7	[5.6–22.6]	46	45.5	[31.1–60.8]	20	19.6	[10.9–32.8]
45–54	44	36.5	[24.1–51.0]	15	12.8	[6.6–23.2]	85	74.9	[63.0–83.9]	14	15.2	[6.7–30.9]
55–64	62	43.7	[32.0–56.1]	31	30.5	[18.8–45.4]	79	57.7	[44.8–69.5]	27	26.9	[16.7–40.3]
≥ 65	59	55.4	[39.6–70.1]	27	27.6	[17.4–40.9]	64	52.6	[37.9–66.8]	25	26.2	[16.0–39.9]
Sex												
Males	66	37.8	[27.1–49.8]	18	10.8	[5.4–20.6]	89	54	[41.5–66.0]	29	19.5	[12.3–29.5]
Females	137	44.7	[34.6–55.3]	66	22.3	[15.2–31.4]	185	57.4	[46.4–67.6]	57	21.9	[15.2–30.6]
Ethnicity												
African	133	41.1	[31.7–51.2]	60	20.3	[13.4–29.5]	160	56.4	[45.6–66.7]	56	21	[15.5–27.9]
White	1	^a^	–	0	^a^	–	2	^a^	–	1	^a^	–
Mixed race	52	42.5	[29.1–57.1]	17	15.9	[7.3–31.3]	54	43.4	[31.7–55.9]	10	6.5	[3.6–11.7]
Indian/Asian	17	54	[26.7–79.2]	7	7.3	[2.2–21.9]	58	64.1	[28.7–88.8]	19	34.5	[12.6–65.8]
Highest Education												
No formal schooling/Gr0–7	122	47.9	[37.0–59.1]	51	23.6	[14.6–35.8]	121	53	[42.3–63.5]	34	19.9	[13.3–28.6]
Grade 8–12 (or equivalent)	76	39.4	[28.5–51.5]	29	14	[8.3–22.7]	137	60.6	[48.4–71.5]	48	22.6	[15.0–32.6]
Higher education	5	^a^	–	4	^a^	–	16	^a^	–	4	^a^	–
Employed												
No	166	44.9	[35.0–55.3]	72	20.2	[13.7–28.9]	207	51.7	[41.2–62.2]	67	19.9	[14.6–26.6]
Yes	37	34	[21.2–49.5]	12	12.2	[5.5–24.9]	67	70.2	[57.2–80.7]	19	24.9	[12.9–42.6]
Urban/Rural residence												
Rural	96	61.5	[51.2–70.8]	40	27.6	[17.4–40.8]	61	40.3	[29.0–52.8]	32	23.5	[16.5–32.5]
Urban	107	32.5	[22.6–44.1]	44	13.5	[7.8–22.4]	213	64.3	[52.4–74.7]	54	19.8	[13.3–28.6]
Province												
Western Cape	42	41.1	[25.5–58.6]	5	8	[2.4–23.3]	37	49.7	[32.9–66.6]	6	6.9	[2.6–16.8]
Eastern Cape	14	29.2	[13.1–52.8]	3	4	[1.2–12.8]	19	60.8	[38.0–79.6]	4	16.4	[4.8–43.1]
Northern Cape	10	28.6	[11.5–55.4]	8	25	[12.1–44.5]	20	51.5	[27.6–74.7]	4	15	[7.1–29.1]
Free State	15	34.1	[15.5–59.3]	6	20.6	[3.1–67.7]	29	69.7	[43.4–87.3]	1	5	[0.9–23.1]
KwaZulu Natal	75	59	[43.8–72.7]	44	29	[18.2–42.8]	103	48.9	[32.7–65.4]	41	26.9	[18.0–38.0]
North West	11	37.3	[21.4–56.5]	6	18.8	[8.5–36.4]	10	40.7	[20.3–64.9]	9	43.9	[21.1–69.6]
Gauteng	16	22.9	[11.1–41.2]	11	17.9	[6.9–39.1]	35	64.9	[42.4–82.3]	14	25.9	[13.3–44.3]
Mpumalanga	4	^a^	–	0	^a^	–	4	^a^	–	0	^a^	–
Limpopo	16	43.9	[19.4–71.8]	1	1.3	[0.2–9.5]	17	67.7	[42.1–85.8]	7	16.4	[4.8–43.3]
Self-reported diagnosis of diabetes												
No previous diabetes diagnosis	152	41.6	[33.1–50.6]	62	17.1	[11.3–25.1]	214	52.7	[43.2–62.0]	69	22.4	[16.6–29.5]
Previous diabetes diagnosis	51	45.2	[29.2–62.4]	22	23	[12.9–37.6]	60	70.3	[56.0–81.4]	17	15.7	[8.5–27.1]
Years since last eye examination												
≤ 2 years	57	39.2	[27.1–52.8]	20	17	[10.0–27.5]	104	60	[46.8–71.9]	25	20.2	[11.1–34.1]
3–5 years	14	43.9	[17.0–75.0]	6	10.2	[3.0–29.3]	25	62.2	[27.8–87.5]	10	25.5	[9.2–53.4]
> 5 years	7	^a^	–	4	^a^	–	18	^a^	–	8	^a^	–
Never	125	44.2	[33.8–55.1]	54	19.8	[13.0–29.1]	127	51.6	[40.3–62.8]	43	18.5	[12.8–26.0]
Do you use eyeglasses or contact lenses to see far away^c^												
Yes	42	38.5	[22.7–57.1]	12	14.5	[7.1–27.6]	79	72.9	[55.9–85.1]	26	30.7	[16.8–49.3]
No	161	43.5	[34.6–52.8]	72	19.4	[12.8–28.2]	192	51.5	[41.7–61.1]	60	18.6	[13.7–24.7]
Do you use eyeglasses or contact lenses to see up close^c^												
Yes	44	35.1	[20.4–53.2]	18	14.4	[7.3–26.4]	98	77	[60.7–87.8]	27	27.8	[15.0–5.7]
No	158	44.8	[35.8–54.2]	66	19.7	[13.1–28.5]	170	49.5	[39.5–59.6]	59	19.3	[14.2–25.7]
In the last 30 days, how difficult was it to see & recognize an object or a person you know across the road (from a distance of about 20 m)? ^c^												
None	114	39.9	[30.5–50.1]	45	17.7	[11.5–26.2]	150	56.4	[46.1–66.3]	42	17.2	[11.3–25.2]
Mild	31	35.6	[21.3–53.0]	23	27.1	[14.8–44.3]	53	60.6	[40.5–77.7]	19	23.9	[14.3–37.0]
Moderate	30	49.1	[32.6–65.9]	8	17	[7.8–33.1]	34	52.7	[35.4–69.4]	10	23.2	[12.5–38.9]
Severe	24	57.7	[32.9–79.1]	5	7.3	[2.5–19.6]	26	50.1	[25.5–74.6]	12	32	[12.9–60.0]
In the last 30 days, how difficult was it to see & recognize an object at arm’s length^c^												
None	116	40.6	[31.0–51.0]	47	18	[11.6–26.9]	142	55.5	[45.1–65.4]	40	17.4	[11.1–26.3]
Mild	32	37	[21.4–55.9]	21	25.8	[14.2–42.2]	61	56.8	[39.4–72.6]	23	24.1	[13.3–39.6]
Moderate	29	47	[31.4–63.2]	10	19.8	[9.4–37.0]	36	56.5	[38.2–73.1]	8	21.4	[11.0–37.5]
Severe	22	53.4	[29.3–76.0]	3	5.2	[1.5–16.1]	25	56.5	[31.0–79.0]	12	31.2	[12.9–58.2]

n, frequency; %, percentage of the frequency; *CI* Confidence Interval; ^a^*n* < 30; ^b^one or more of the following: loss of peripheral vision, difficulty driving at night, double vision, difficulty in distinguishing colours, straight lines looking wavy, and sensitivity to glare; ^c^totals per category do not always add to overall total due to missing data and non-response (the number of missing observations for the last 4 variables; using visual aids to see far away, using visual aids to see up close, difficulty in seeing objects from across the road and difficulty in seeing objects at arm’s length; were 3, 7, 16, and 15 respectively)

**Table 4:** Factors associated with vision loss

	Univariate logistic regression			Multiple logistic regression^b^		
	Crude OR	95% CI (Crude OR)	*p*-value	AOR	95% CI (AOR)	*p*-value
Age (years)						
15–44	ref	–	–	ref	–	–
45–54	3.79^a^	[2.44–5.87]	< 0.001	2.99 ^a^	[1.93–4.62]	< 0.001
55–64	5.71 ^a^	[3.5–9.31]	< 0.001	5.78 ^a^	[3.73–8.98]	< 0.001
≥ 65	5.00 ^a^	[2.97–8.43]	< 0.001	5.12 ^a^	[3.16–8.27]	< 0.001
Sex						
Males	ref	–	–	ref	–	–
Females	1.57 ^a^	[1.17–2.1]	0.003	1.50 ^a^	[1.08–2.08]	0.016
Ethnicity						
African	ref	–	–	ref	–	–
White	0.23 ^a^	[0.07–0.74]	0.014	0.11 ^a^	[0.02–0.56]	0.007
Mixed race	0.94	[0.58–1.51]	0.801	0.82	[0.43–1.56]	0.541
Indian/Asian	4.09 ^a^	[2.5–6.71]	< 0.001	1.74	[0.88–3.45]	0.112
Highest Education						
No formal schooling/Gr0–7	ref	–	–	ref	–	–
Grade 8–12 (or equivalent)	0.46^a^	[0.33–0.63]	< 0.001	0.71 ^a^	[0.52–0.97]	0.031
Higher education	0.38 ^a^	[0.19–0.75]	0.006	0.48	[0.22–1.03]	0.059
Employed						
No	ref	–	–			
Yes	0.77	[0.51–1.16]	0.207			
Urban or rural residence						
Rural	ref	–	–			
Urban	1.19	[0.81–1.75]	0.382			
Province						
Western Cape	ref	–	–	ref	–	–
Eastern Cape	0.79	[0.42–1.49]	0.461	0.63	[0.28–1.41]	0.263
Northern Cape	0.73	[0.34–1.57]	0.419	0.62	[0.28–1.37]	0.240
Free State	1.30	[0.58–2.9]	0.526	1.07	[0.42–2.71]	0.894
KwaZulu Natal	2.52 ^a^	[1.44–4.4]	0.001	1.86	[0.88–3.9]	0.102
North West	0.63	[0.29–1.36]	0.239	0.43	[0.17–1.11]	0.082
Gauteng	1.19	[0.59–2.38]	0.631	1.07	[0.5–2.32]	0.855
Mpumalanga	0.16 ^a^	[0.06–0.45]	0.001	0.12 ^a^	[0.04–0.36]	< 0.001
Limpopo	0.92	[0.45–1.87]	0.818	0.78	[0.26–2.35]	0.662
Diabetes diagnosis						
No previous diagnosis of diabetes	Ref	–	–	ref	–	–
Previous diagnosis of diabetes	5.07 ^a^	[3.32–7.73]	< 0.001	2.28 ^a^	[1.38–3.75]	0.001
Years since last eye examination						
≤ 2 years	Ref	–	–	ref	–	–
3–5 years	0.99	[0.5–1.97]	0.980	0.85	[0.39–1.88]	0.690
> 5 years	0.63	[0.31–1.28]	0.199	0.66	[0.3–1.42]	0.285
Never	0.54 ^a^	[0.37–0.79]	0.001	0.56 ^a^	[0.38–0.82]	0.003

ref, reference; ^a^statistically significant; *OR* Odds ratio, *AOR* Adjusted odds ratio, *CI* Confidence Interval. ^b^The following variables were included in the multiple logistic regression model: age group, sex, ethnicity, education, province, diabetes diagnosis, and number of years since last eye examination

The multiple logistic regression model (see Table 4) included all the variables found to have a significant association with vision loss in the univariate logistic regression; namely, age group, sex, ethnicity, education, province, diabetes diagnosis, and number of years since last eye examination. In the multiple logistic regression, older age (45–54 years OR = 2.99, *p* < 0.001, 55–64 years OR = 5.78, *p* < 0.001 and ≥ 65 years OR = 5.12, *p* < 0.001 compared with 15–44 years), female sex (OR = 1.50, *p* = 0.016), and previous diabetes diagnosis (OR = 2.28, *p* = 0.001) were significantly associated with increased odds of vision loss. Secondary school education (OR = 0.71, *p* = 0.031 compared with primary school or no formal education), white ethnicity (OR = 0.11, *p* = 0.007 compared with African ethnicity), residing in Mpumalanga province (OR = 0.12, *p* < 0.001) and having never had an eye examination (OR = 0.56, *p* = 0.003 compared with having had an eye examination within the preceding 2 years) were significantly associated with reduced odds of vision loss.

## Discussion

In this study, we investigated the prevalence of vision loss among South Africans using data from the South African National Health and Nutrition Examination Survey (SANHANES-1), a population-based bio-behavioural national survey conducted in 2011–2012. The results showed that nearly one in ten of all the participants had vision loss. In the multiple logistic regression, which adjusted for age, sex, ethnicity, education, province, previous diabetes diagnosis and number of years since last eye examination, older age, female sex, and previous diabetes diagnosis were found to be associated with higher odds of vision loss whereas, white ethnicity, formal education (completion of secondary or tertiary education), residing in Mpumalanga province, and having never had an eye examination were significantly associated with lower odds of vision loss.

Naturally, aging presents with various structural and functional changes that make one susceptible to ocular morbidity. As individuals age, eye diseases (cataract, glaucoma, age-related macular degeneration, and presbyopia) occur and consequently lead to vision loss (if untreated) [17–19]. Globally, the prevalence of vision loss increases with age [1, 2]. Thus, with the world population growth on the ascendency and the elderly having a longer lifespan, eye care needs for the elderly is expected to rise. Our result is consistent with studies among Nigerian [38], Chinese American [47], and Iranian [48] populations, which shows older age to be significantly associated with higher odds of vision loss. Similar studies in various provinces in South Africa have shown similar findings [27, 41]. Besides differences in the methodology of the various studies, there is a general positive association between increasing age and the odds of having vision loss.

In this study, female sex was noted to be significantly associated with the prevalence of vision loss. Generally, women are known to be at increased risk of vision loss than men [39, 49, 50]. Women’s increased susceptibility to vision loss may be partly due to their higher life expectancy than men [51, 52]. Although the regression analysis adjusted for age, the adjustment may have been partial, because the age-groups used may have needed to be more finely disaggregated to distinguish participants older than 65 years. Evidence from studies show that variations in the composition of hormones and chromosomes in men and women account for the differences in longevity [53]. For instance, women appear to have more subcutaneous fat, whereas men have more visceral fat, which is predictive of cardiovascular disease [53]. This distribution is impacted by oestrogen, and the additional X chromosome predominantly found in women. Thus, with women having a longer lifespan than men in South Africa (67.7 years as compared to 61.5 years), it is expected that they may have a higher risk of developing ocular morbidities than men [35]. The gender disparity regarding access to medical care services, especially in some developing countries, could account for the higher prevalence of vision loss in women [54]. Thus, the existence of socio-economic and cultural disparities between men and women in developing countries contribute to women having higher prevalence of vision loss. For instance, women may have restricted movement, their eye care needs may not be deemed as urgent compared to males, and they may not have the financial ability to pay for eye care services. These situations deny women of the opportunity to access healthcare, and thus, makes it difficult to detect and prevent ocular diseases among women in the early stages [55]. This finding is consistent with studies in South Africa [32, 41], Nigeria [38, 40, 54], Iran [48], and Malaysia [56]. This behoves policymakers to create awareness and avenues for women to engage in meaningful and productive ventures with reasonable remuneration. Health education and promotion can be tailored for women, to enable them to be more proactive in their healthcare seeking behaviours, empower them to make informed decisions on matters pertaining to their health and increase their accessibility to health care services.

Interestingly, higher education levels (completion of secondary school or tertiary education) remained significantly associated with a decreased prevalence of vision loss in this study. Notably, not only does educational knowledge shape an individual’s employment prospects (i.e., better remuneration, favorable working conditions, and better health-related benefits), but it also empowers them to make informed decisions regarding their health (i.e., having a higher demand for good sight and easy accessibility to eye care services) than the people without formal education [57, 58]. On the contrary, not having access to formal education may be associated with a higher use of traditional medicine [59]. This means has proven unsuccessful over time and is known to exacerbate the burden of vision loss. Furthermore, it is worth mentioning that people who do not have access to formal education are more likely to have difficulty obtaining healthcare, as poverty impedes both access to education and access to healthcare [27, 60]. A study by Gilbert et al. in Nigeria reported that majority of the residents without formal education in rural communities resorted to couching (a traditional treatment of cataract by dislocating the cataractous lens into either the anterior segment or the posterior segment with no intraocular lens implantation), which rendered close to half of the individuals who had undergone couching blind [59]. Our finding is consistent with studies in India [61, 62], China [63, 64], and Nepal [65], which indicates the need for the institution of practical measures to promote health education regarding ocular health, especially in rural communities across the nation.

Despite the government’s efforts in reducing inequity and improving access to health care, there still exist disparities in health status and accessibility to healthcare services, including eye care in South Africa [66]. Most eye care providers (ophthalmologists and optometrists) in South Africa are with the private sector in the urban areas of the provinces, leaving minimal numbers in the public sector to cater for the majority of the populace [67]. Hence, with a considerable proportion of the people in poverty living in rural areas [68], coupled with the differences in the distribution of healthcare personnel, and the unavailability of eye care service providers in the rural areas as compared to the urban communities, make access to health care services extremely challenging and thus, may lead to increased prevalence of vision loss in the rural areas [69–72]. Furthermore, blurred vision and difficulty reading were the main contributors to vision loss in our study. This could be attributable to refractive errors and presbyopia, which could be easily corrected with prescription glasses [1–3, 41, 42, 71, 73, 74]. However, barriers such as the cost of transport and high prices of eye care services, as well as prescription spectacles, deter the people in poverty from patronizing the services of eye care professionals [75]. Therefore, a timely intervention by the government and stakeholders of healthcare through the training of adequate eye health workers and the provision of the necessary health equipment will promote the efficient and effective utilization of eye care services. This measure will decrease the burden of vision loss and its impact on the quality of life of affected individuals.

In recent times, the National Prevention of Blindness Program has outlined some health guidelines aimed at coordinating and providing support towards blindness prevention, improving access to primary eye care, and promoting the rights of the blind in South Africa [31]. These eye health guidelines are mainly curative, and thus, there is an absence of an integrated eye health promotional policy and a dedicated directorate to monitor and evaluate eye health promotional activities in South Africa [76]. Although the government collaborates with both local and international agencies to address these gaps, these collaborations are short-lived due to the financial constraints involved. Further, a prospective study by Lilian et al., which assessed the primary eye care services in three South African districts, showed the need for introducing health system strengthening (HSS) package in primary eye care delivery. The study revealed that HSS enhanced organizational care and clinical practice [77]. Therefore, to curb the increasing occurrence of people going needlessly blind or having visual impairments, it is imperative that policymakers institute measures that aim to integrate ocular health promotional activities in the primary health care system.

Our study identified self-reported visual difficulty as a vital component of vision loss. Thus, a considerable number of participants (with or without spectacle use) had vision loss. This challenge could partly result from the unavailability of service providers within the needed catchment areas as well as inadequate education about the relevance of optical correction in optimizing sight [69–71]. Hence, participants resort to adaptive means of vision enhancement such as increasing illumination, increasing of font size, and reading at arm’s length, which could account for the low utilization of spectacles. This explains why most of the participants had no difficulty in recognizing objects at arm’s length or persons across the street. This finding is consistent with a study by Naidoo et al., which aimed at investigating the prevalence of self-reported vision difficulty in some regions of South Africa [42]. Therefore, appropriate implementation of cost-effective eye health screening programs by policymakers is paramount in reducing the burden of vision loss, especially in deprived regions in South Africa.

Moreover, the absence of simple, effective screening equipment for diabetic retinopathy in the various primary healthcare in South Africa could account for the four-fold increase in odds of vision loss among diabetics in this study [76, 78, 79]. Thus, the majority of patients only sought medical care once retinopathies have already developed, and vision affected considerably [80]. A pilot screening study with a mobile fundus camera in Cape Town proved useful as a single technician could screen about 10,000 patients in a year [80]. However, a study by Tu et al. reported poor cost-effectiveness of digital photography screening among individuals with diabetic retinopathy in the United Kingdom, and this primarily resulted from poor compliance rates [81]. Hence, the use of a digital fundus camera as a screening tool is not wholly accepted by practitioners. Therefore, with the increasing trend of diabetes prevalence in South Africa [82], the provision of appropriate health supplies coupled with established screening protocol and referral system will ensure effective monitoring of individuals with diabetes. These measures will enable early detection and therapeutic management of blinding conditions and, consequently, reduce the burden of vision loss caused by diabetes.

The strength of this study lies in the fact that it uses the most recent nationally representative data of all ages, which provides adequate information on vision loss in South Africa. Furthermore, this study provides an appraisal of the efficiency of the existing structures and policies on eye care services to inform amendment and improvement in strategies regarding vision loss in South Africa. A major limitation of our study is that SANHANES-1; due to a large number of physical examinations and biomarker assessments being conducted did not perform a comprehensive ocular examination to determine the specific cause of vision loss. However, from the reported symptoms/signs, one could hypothesize or project the potential causes of vision loss. Again, it is worth mentioning that the subsample of participants whose vision was assessed is considerably smaller than the total sample. The subsample had a slightly higher proportion of female sex than the total sample, which may have introduced selection bias. Of note, another limitation of our study has to do with the use of relatively older research data. Nonetheless, this vital data has been the only population-based national health survey conducted in South Africa since 2011 that provides data on vision loss. Hence, our data primarily serves as a baseline for investigating vision loss and its associated factors in subsequent population-based studies in South Africa.

## Conclusion

In summary, almost one in ten South Africans had vision loss. Older age, female sex, and having a previous diagnosis of diabetes were associated with higher odds of vision loss. On the contrary, living in Mpumalanga province, having secondary or tertiary education, and white ethnicity significantly reduced the odds of vision loss. These findings call on policymakers, government officials, and healthcare administrators to adopt strategies targeted at reducing barriers to eye care services such as improving accessibility, subsidizing the cost of health services, training of eye care professionals, and siting of eye care facilities in areas with low coverage of ocular health services. Furthermore, the findings suggest the need for health education and health promotion interventions that are targeted and tailored to groups of individuals that have a higher risk of vision loss, in order to increase awareness and promote eye care seeking behaviours. These strategies will promote early detection and management of blinding conditions and thereby, decrease the burden of vision loss in the country.

## Data Availability

The dataset(s) supporting the conclusions of this article is (are) available on request from the HSRC. The SANHANES data are available through registered access from the Human Sciences Research Council’s (HSRC) data repository at http://curation.hsrc.ac.za/Datasets-XKAHAA.phtml.

## Abbreviations

AMD: Age-related Macular Degeneration
DFID: United Kingdom Department for International Development
HSRC: Human Sciences Research Council
REC: Research Ethics Committee
SANHANES: South African National Health and Nutrition Examination Survey
WHO: World Health Organization
HSS: Health System Strengthening
PVA: Presenting Visual Acuity
